# Potential relation between soluble growth differentiation factor-15 and testosterone deficiency in male patients with coronary artery disease

**DOI:** 10.1186/s12933-019-0823-3

**Published:** 2019-02-28

**Authors:** Huan Liu, Yongnan Lyu, Di Li, Yan Cui, Yun Huang, Wen Dai, Yan Li

**Affiliations:** 1 Dept of Clinical Laboratory, Wuhan Univ, Renmin Hospital, Jiefang Road 238, Wuhan, 430060 Hubei China; 2 Dept of Cardiology, Wuhan Univ, Renmin Hospital, Jiefang Road 238, Wuhan, 430060 Hubei China

**Keywords:** Growth differentiation factor-15, Testosterone, Coronary artery disease

## Abstract

**Background:**

There is a mutual interaction between inflammation and endocrine disorders in the development of coronary artery disease (CAD). Growth differentiation factor-15 (GDF-15) is associated with CAD, and the effects of testosterone on CAD as reported in literature have been considered as anti-atherosclerotic. The present study aimed to examine the possible association between serum GDF-15 and testosterone in male CAD patients.

**Methods:**

GDF-15 and testosterone concentrations were determined in blood samples of 426 male patients with CAD and 220 male controls. Serum concentrations of hs-CRP, and other baseline characteristics were also measured.

**Results:**

Serum levels of GDF-15 were higher in CAD patients when compared to controls, and testosterone concentrations were lower (*p *< 0.001). Patients with low testosterone levels had higher concentrations of GDF-15 (*p *< 0.001). In stratified analyses, inverse relations between GDF-15 levels and testosterone were noted for almost all strata, stratified by categories of hs-CRP, leukocytes, neutrophils, neutrophil to lymphocyte ratio, glucose, HDL-c, and LDL-c, and whether had hypertension, diabetes, and underwent percutaneous coronary intervention (PCI). Furthermore, in the linear regression models with bootstrap resampling with 1000 replications, high GDF-15 levels were independently associated with testosterone deficiency in male patients with CAD.

**Conclusions:**

In male patients with CAD, high GDF-15 levels were associated with testosterone deficiency. These results support that upregulation of GDF-15 in the presence of low testosterone levels during CAD progression is a potential mechanism by which GDF-15 affects CAD.

**Electronic supplementary material:**

The online version of this article (10.1186/s12933-019-0823-3) contains supplementary material, which is available to authorized users.

## Background

Coronary artery disease (CAD) is considered one of the leading causes of death within the United States and globally in light of rapid epidemiological transitions over the past 2 decades, bringing a great economic burden to the society. Thus, primary prevention of CAD is critical, and attention has turned toward screening for CAD risk by identifying “high-risk” individuals. Atherosclerosis is the early stage of CAD, and nowadays the concept of atherosclerosis has been changed into the disorders of various inflammation [1–3]. The recent availability of novel biomarkers that better reflect the progression and the severity of CAD, notably growth differentiation factor-15 (GDF-15), has emerged as a promising novel biomarker for risk assessment and prognostic evaluation.

CAD affects men and women differently. For men, a low testosterone status is associated with an increase in atherosclerosis and cardiovascular risk, and the risk of CAD rises dramatically after 50 years of age [4]. It has been hypothesized that lower levels of endogenous androgens that occur as a consequence of the increased age might mediate the increased CAD risk later in life in older men. This theory has been supported by observational studies that demonstrate associations between lower androgen and CAD risk factors, including elevated high sensitivity C-reactive protein (hs-CRP), arterial stiffness, adiposity, insulin resistance, and beta-cell failure [5–8]. Furthermore, several studies demonstrated that testosterone could directly regulate the expression of GDF-15 in some tumor cells [9, 10], indicating a potential association between sex hormones and GDF-15. Given the relations between testosterone deficiency and the risk of CAD, and the strong association between GDF-15 and CAD. We hypothesize that higher circulating levels of GDF-15 are associated with a greater prevalence of testosterone deficiency, and that is a potential mechanism by which GDF-15 affect CAD. Thus, to verify our hypothesis, we compared GDF-15 levels across different testosterone tertiles and analyzed the association of GDF-15 with testosterone.

## Methods

### Study design and objective

This was a case–control study to evaluate the potential association between serum GDF-15 and testosterone in male CAD patients. The protocol was approved by the Medical Ethics Review Committee of Wuhan Univ, Renmin Hospital. All subjects provided written informed consent (2018 K-C161).

### Sample

All consecutive patients who underwent elective coronary angiography for suspected CAD at Renmin Hospital of Wuhan University from July 2016 to December 2017 were eligible for the study. Individuals with other severe illnesses, such as infectious disease, pulmonary edema, chronic renal function, acute kidney injury and so on or those receiving thrombolysis treatment and hormone replace therapy treatment were excluded. A final total of 646 participants were enrolled. CAD was defined as > 50% occlusion of at least one major coronary artery, as determined by coronary angiography, and those at rest or inducible ischemia on exercise or pharmacologic stress testing with 30% to 50% occlusion of at least one major coronary artery. While those with completely normal coronary arteries and with less than 50% occlusion in all coronary arteries (free of significant stenosis) constituted the control group. According to the diagnostic standard, 426 patients were recruited as CAD, and 220 patients were enrolled as controls.

### Study definitions

AMI was defined as recommended in current guidelines [11]. Hypertension was defined according to World Health Organization (WHO) criteria [12] as average systolic blood pressure 140 mmHg, diastolic blood pressure 90 mmHg, or use of antihypertensive medication. Diabetes was defined according to Anand’s [13], in brief, a fasting glucose level 126 mg/dL (7.0 mmol/L), non fasting glucose 200 mg/dL (11.1 mmol/L), or self-reported diabetes coupled with the use of any glucose-lowering medication.

### Sample preparation

Patients with AMI were generally emergency patients and their blood samples were collected immediately after hospitalization. For others, venipuncture was performed in the morning after an overnight fast [14] and before pharmacotherapy. A sample of 3 mL of blood was collected from the patient’s median cubital vein into a tube. Blood samples were centrifuged after incubation at room temperature for 15 min. Then, the serum was gathered and stored at − 70 °C until measurement.

### Laboratory analyses

Serum concentrations of total cholesterol (TC), triglyceride (TG), high-density lipoprotein cholesterol (HDL-c), low-density lipoprotein cholesterol (LDL-c) and, uric acid (UA), and glucose were measured using an automatic biochemistry analyzer ADVIA 2400 through enzymatic methods (Siemens, Germany). Blood leukocytes and neutrophils were detected by Sysmex XN-20 (Kobe, Japan). And the detection method of hs-CRP is a polyethylene glycol (PEG) enhanced immunoturbidimetric assay.

To assess the soluble form of human GDF-15 levels in crude serum, we adopted commercial enzyme-linked immunosorbent assay (ELISA) kits purchased from R&D (Quantikine, R&D Systems, USA) (Catalog Number: DY957, DY008) with intra- and inter-assay coefficient of variation < 6% and 2.8%, respectively. The range of values detected by this assay was 7.8–500 pg/mL, and the samples has been diluted 10 times for testing. All the measurement of plasma GDF-15 was performed in duplicate for each sample. The principle of this kit was indirect sandwich ELISA-based technique utilizing capture antibodies and biotinylated detection antibodies for capture and detection purposes, respectively.

### Statistical analysis

Statistical analysis was performed with SPSS version 22.0 (IBM, NY, USA) and figures were made with GraphPad Prism V.6.0 (GraphPad Software, Inc, La Jolla, California, USA). Normally distributed continuous variables were presented as mean ± SD and compared by Student *t* test. Variables with skewed distributions were expressed as median (interquartile range [IQR]) and compared using the Mann–Whitney test. Categorical variables were presented as percentage (%) and compared using the Chi square test. Because the distribution of values for age, hs-CRP, neutrophil to lymphocyte ratio, and glucose were strongly skewed, we transformed them to the log_10_ scale for the comparison of different groups across testosterone tertiles. And we also transformed GDF-15 levels to the log10 scale for stratified analysis and covariance analysis, and adjusted for hypertension, diabetes, and age. The associations between GDF-15 and testosterone were evaluated using multivariate linear regression models with bootstrap resampling after adjustment for age, hs-CRP, WBC, NEU, neutrophil to lymphocyte ratio, glucose, TC,TG, HDL-c, and LDL-c. A two-sided *p* value of < 0.05 was considered statistically significant.

## Results

### Characteristics of controls and male patients with CAD

Baseline characteristics of all patients are summarized in Table 1. Age and most other cardiovascular risk factors, such as hypertension, and diabetes did not differ between controls and CAD patients. In the analysis of lipid parameters, CAD patients had an unfavorable lipid profile, including higher concentrations of TC, TG, LDL-c, and lower levels of HDL-c. Besides, CAD patients had a stronger inflammatory reaction, including higher levels of hs-CRP, and higher number of blood leukocytes, and neutrophils in comparison to controls. The higher leukocyte counts in the blood of CAD patients can be attributed to the higher number of neutrophils. Moreover, CAD patients had higher levels of GDF-15 and lower levels of testosterone, compared with controls (*p *< 0.001). To further investigate whether the changes of testosterone and GDF-15 were associated with disease severity. We analyzed them among stable angina (SA), unstable angina (UA) and acute myocardial infarction (AMI) patients. Our results showed that serum GDF-15 levels tend to increase, while serum testosterone levels tend to decrease among these three groups (Additional file 1: Table S1).

**Table 1:** Characteristics of biochemical data between controls and matched CAD patients

Characteristics	Controls (n = 220)	CAD (n = 426)	*p* value
Age (years)	62.00 (53.00–69.00)	61.00 (54.00–67.00)	0.178
Diabetes (%)	20.19	27.45	0.096
Hypertension (%)	48.40	51.6	0.650
hs-CRP (mg/dL)	0.46 (0.20–0.86)	2.28 (0.50–10.54)	< 0.001
WBC (10^9^/L)	5.55 (4.83–6.45)	7.37 (5.93–9.06)	< 0.001
NEU (10^9^/L)	3.57 (2.74–4.93)	4.69 (3.50–6.62)	< 0.001
Glucose (mmol/L)	5.45 (5.17–5.91)	5.80 (5.07–7.10)	< 0.001
TC (mmol/L)	3.90 (3.34–4.55)	4.34 (3.95–4.66)	< 0.001
TG (mmol/L)	1.09 (0.81–1.41)	1.45 (1.04–2.11)	< 0.001
HDL-c (mmol/L)	1.14 (1.01–1.30)	0.88 (0.76–1.07)	< 0.001
LDL-c (mmol/L)	2.13(1.64–2.71)	2.38 (2.04–2.64)	< 0.001
Testosterone (ng/dL)	466.27 (364.61–533.00)	292.02 (206.91–391.94)	< 0.001
GDF-15 (pg/mL)	347.99 (256.45–440.84)	775.07 (446.12–1113.19)	< 0.001

Data are expressed as median (25th percentile–75th percentile); nominal data are given as percentages. Chi square (nominal data) or Mann–Whitney-U test (interval data) were performed to compare controls and CAD patients

*hs*-*CRP* high-sensitivity C-reactive protein, *WBC* white blood cell, *NEU* neutrophil, *TC* total cholesterol, *TG* triglycerides, *HDL*-*c* high-density lipoprotein cholesterol, *LDL*-*c* low-density lipoprotein cholesterol, *GDF*-*15* growth differentiation factor-15

### Baseline characteristics stratified by testosterone tertiles

**Fig. 1:**
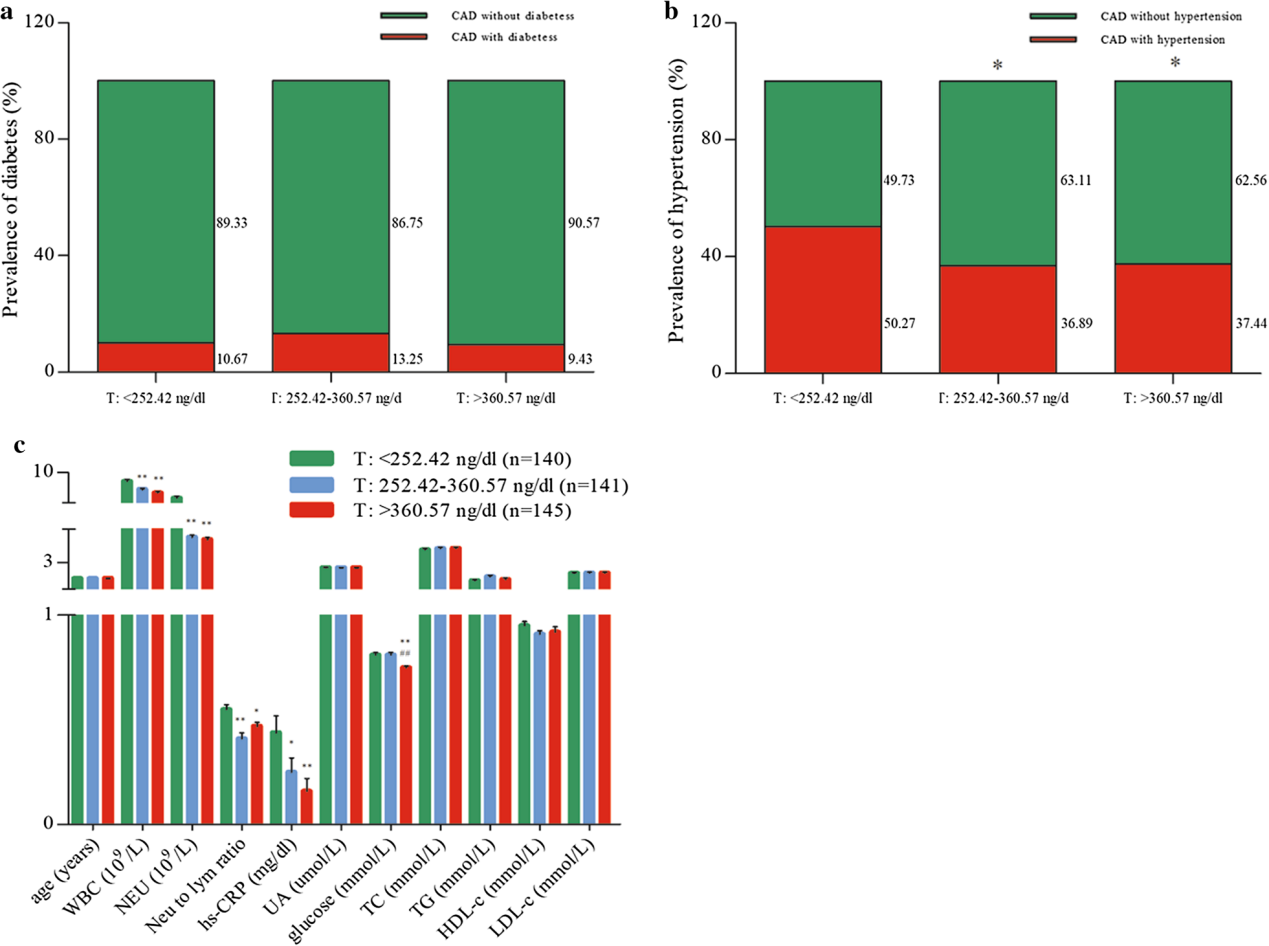
Baseline characteristics of 426 male patients with CAD according to testosterone tertiles. **a** Comparison of the prevalence of diabetes in CAD patients. **b** Comparison of the prevalence of hypertension in CAD patients. **c** Age, numbers of blood leukocytes and neutrophils, neutrophil to lymphocyte ratio, and serum concentrations of hs-CRP, UA, glucose, TC, TG, HDL-c, LDL-c, and GDF-15 across different testosterone tertiles. Data are shown as mean ± standard deviation. *Compared with CAD patients with testosterone levels lower than 254.90 ng/dL, *p *< 0.05; **compared with CAD patients with testosterone levels lower than 254.90 ng/dL, *p *< 0.001; ^##^compared with CAD patients with testosterone levels higher than 254.90 ng/dL, and lower than 360.49 ng/dL, *p *< 0.001

Baseline characteristics of the 426 male patients stratified by testosterone tertiles are presented in Fig. 1. The median age of the study cohort was 61.00 years (IQR 54.00–67.00 years). At baseline, age, diabetes, levels of TC, TG, HDL-c, LDL-c, and UA did not differ across the different testosterone groups. CAD patients with low testosterone levels had a more unfavorable inflammatory reaction compared to patients with high testosterone levels as observed by higher levels of hs-CRP (T1: 0.44 ± 0.97 mg/dL vs. T2: 0.25 ± 0.81 mg/dL, *p *< 0.001; T1: 0.44 ± 0.97 mg/dL vs. T3: 0.16 ± 0.74 mg/dL, *p *< 0.05). Moreover, the measurements showed a consistent direction of effect over all groups. Patients with low levels of testosterone had higher numbers of blood leukocytes and neutrophils when compared to patients with high testosterone concentrations, and showed an direction of effect over the three groups (for leukocytes, T1: 8.66 ± 3.40 10^9^/L vs. T2: 7.47 ± 2.02 10^9^/L, *p *< 0.001; T1: 8.66 ± 3.40 10^9^/L vs. T3: 6.98 ± 1.97 10^9^/L, *p *< 0.001, for neutrophils, T1: 6.20 ± 3.30 10^9^/L vs. T2: 4.89 ± 2.10 10^9^/L, *p *< 0.001; T1: 6.20 ± 3.30 10^9^/L vs. T3: 4.72 ± 1.98, *p *< 0.001). The neutrophil-to-lymphocyte ratio, which is a strong predictor for future adverse events and reflects inflammatory status in CAD patients was lowest in patients with high testosterone levels (T1: 0.55 ± 0.29 vs. T2: 0.41 ± 0.33, *p *< 0.001; T1: 0.55 ± 0.29 vs. T3: 0.47 ± 0.23, *p *< 0.05).

### Comparison means of GDF-15 across different testosterone categories

**Fig. 2:**
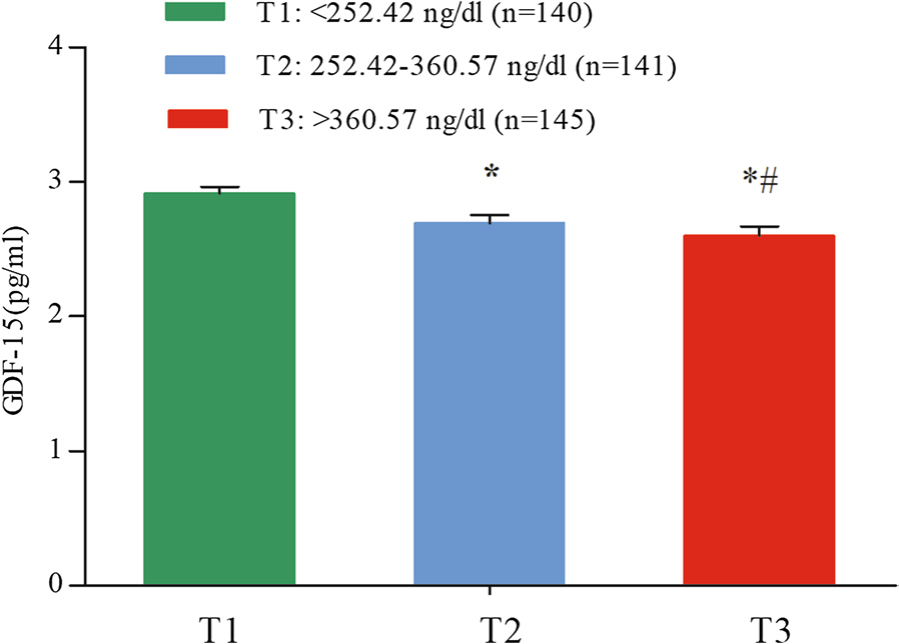
Serum concentrations of GDF-15 among different testosterone tertiles. GDF-15 was log10 transformed. The data is presented as the mean value ± SEM, covariance analysis were used to compare the levels of GDF-15 across testosterone groups. Compared with the group with testosterone levels lower than 254.90 ng/dL, *p < 0.05; compared with the group with testosterone levels higher than 254.90 ng/dL, and lower than 360.49 ng/dL, ^#^*p* < 0.05

As shown in Fig. 2, after adjusting for age, hypertension, and diabetes, GDF-15 decreased significantly across testosterone categories in covariance analysis (*p *< 0.001). The levels of serum GDF-15 in patients with testosterone levels lower than 254.90 ng/dL was 2.91 ± 0.05 pg/mL, which decreased to 2.69 ± 0.06 pg/mL in patients with testosterone levels higher than 254.90 ng/dL, and lower than 360.49 ng/dL (*p *< 0.05). Furthermore, the levels of serum GDF-15 in patients with testosterone levels higher than 360.49 ng/dL (2.60 ± 0.07 ng/dL) were significantly lower than the other groups (all *p *< 0.05).

### Stratified analyses

Table 2 shows associations between testosterone and GDF-15 levels obtained using linear regression models on log_10_(GDF-15) in patients with CAD, stratified by categories of hs-CRP, leukocytes, neutrophils, neutrophil to lymphocyte ratio, glucose, HDL-c, and LDL-c, with adjustment for hypertension, diabetes, and age. We also analyzed the association of GDF-15 with testosterone in CAD patients with hypertension, diabetes, and underwent percutaneous coronary intervention (PCI) or not, respectively. Linear trend tests gave statistically significant results for the total sample (*p* < 0.001) and for most of the strata. In patients underwent PCI and had higher levels of hs-CRP, neutrophils, neutrophil to lymphocyte ratio, glucose, and lower levels of HDL-c, testosterone correlated significantly with GDF-15. Inverse relations between GDF-15 levels and testosterone were noted for almost all strata stratified by the median of leukocytes, UA and LDL-c (all *p *< 0.05). Furthermore, in CAD patients with hypertension, and diabetes or not, testosterone correlated significantly with GDF-15.

**Table 2:** Adjusted association between testosterone and GDF-15 levels in the total sample and across categories of hs-CRP, WBC, NEU, neutrophil to lymphocyte ratio, glucose, HDL-c, and LDL-c and whether had hypertension, diabetes, and underwent PCI subgroups

Variable	Unstandardized coefficient (B)	Standardized (B)	95% CI for B	*P* for trend
Total	− 41.598	− 0.145	− 62.520 to − 20.676	< 0.001
Hypertension				
CAD without hypertension	− 55.676	− 0.182	− 103.221 to − 8.132	0.022
CAD with hypertension	− 50.098	− 0.177	− 86.648 to − 13.548	0.007
Diabetes				
CAD without diabetes	− 42.299	− 0.149	− 73.706 to − 10.893	0.008
CAD with diabetes	− 99.626	− 0.298	− 173.457 to − 25.795	0.009
PCI				
CAD without PCI	− 42.809	− 0.158	− 90.698 to 5.079	0.079
CAD with PCI	− 67.819	− 0.215	− 105.559 to − 30.080	< 0.001
hs-CRP				
Lower value (< 1.85 mg/dL)	− 30.174	− 0.121	− 69.087 to 8.738	0.128
Higher value (> 1.85 mg/dL)	− 64.611	− 0.201	− 114.637 to − 14.585	0.012
WBC				
Lower value (< 7.25 (10^9^/L))	− 42.226	− 0.162	− 78.957 to − 5.496	0.024
Higher value (> 7.25 (10^9^/L))	− 60.663	− 0.178	− 109.975 to − 11.352	0.016
NEU				
Lower value (< 4.62 (10^9^/L))	− 31.812	− 0.119	− 69.644 to 6.019	0.099
Higher value (> 4.62 (10^9^/L))	− 76.342	− 0.238	− 121.420 to − 31.265	0.001
Neutrophil to lymphocyte ratio				
Lower value (< 2.83)	− 25.081	− 0.094	− 63.680 to 13.517	0.201
Higher value (> 2.83)	− 86.434	− 0.267	− 130.118 to − 42.750	< 0.001
Glucose				
Lower value (< 5.58 mmol/L)	− 26.029	− 0.099	− 65.959 to 13.901	0.200
Higher value (> 5.58 mmol/L)	− 76.395	− 0.234	− 119.955 to − 32.834	0.001
UA				
Lower value (< 388 umol/L)	− 67.095	− 0.214	− 111.406 to − 22.785	0.003
Higher value (> 388 umol/L)	− 52.117	− 0.194	− 92.312 to − 11.922	0.011
HDL-c				
Lower value (< 0.91 mmol/L)	− 56.000	− 0.190	− 98.395 to − 13.604	0.010
Higher value (> 0.91 mmol/L)	− 40.376	− 0.135	− 86.511 to 5.759	0.086
LDL-c				
Lower value (< 2.16 mmol/L)	− 55.056	− 0.191	− 98.330 to − 11.782	0.013
Higher value (> 2.16 mmol/L)	− 49.148	− 0.159	− 95.688 to − 2.607	0.039

*hs*-*CRP* high-sensitivity C-reactive protein, *WBC* white blood cell, *NEU* neutrophil, *UA* uric acid, *HDL*-*c* high-density lipoprotein cholesterol, *LDL*-*c* low-density lipoprotein cholesterol, *GDF*-*15* growth differentiation factor-15, *PCI* percutaneous coronary intervention

### Multivariate linear regression models

Linear regression models with bootstrap resampling with 1000 replications were implemented as the primary analysis model (Table 3), and testosterone correlated significantly with GDF-15 when adjusting for age, hs-CRP, WBC, NEU, neutrophil to lymphocyte ratio, glucose, TC,TG, HDL-c, and LDL-c (β-coefficient = − 0.044, *p *= 0.003).

**Table 3:** Multivariate linear regression analyses

variables	Unstandardized coefficient (B)	95% CI for B	*p* for trend
Age	1.057	− 0.926 to 3.245	0.297
GDF-15	− 0.044	− 0.074 to − 0.012	0.003
hs-CRP	− 0.392	− 1.449 to 0.880	0.406
WBC	5.608	− 23.031 to 29.967	0.669
NEU	− 17.452	− 43.907 to 14.871	0.234
Neutrophil to lymphocyte ratio	3.093	− 5.705 to 12.823	0.469
Glucose	− 6.720	− 15.827 to − 3.553	0.167
UA	− 0.007	− 0.218 to 0.188	0.943
TC	− 4.534	− 59.304 to 51.261	0.876
TG	− 11.276	− 41.005 to 17.363	0.411
HDL-c	− 0.033	− 134.710 to 138.002	0.999
LDL-c	17.199	− 37.906 to 71.060	0.501

*hs*-*CRP* high-sensitivity C-reactive protein, *WBC* white blood cell, *NEU* neutrophil, *UA* uric acid, *TC* total cholesterol, *TG* triglycerides, *HDL*-*c* high-density lipoprotein cholesterol, *LDL*-*c* low-density lipoprotein cholesterol, *GDF*-*15* growth differentiation factor-15

## Discussion

This retrospective study was the first to evaluate the potential association between GDF-15 and testosterone in male patients with CAD. We observed that GDF-15 levels increased as testosterone decrease after adjusting for age, hypertension, and diabetes. Inverse relations between GDF-15 levels and testosterone were noted for almost all strata, stratified by categories of CAD risk factors, such as hs-CRP, leukocytes, neutrophils, neutrophil to lymphocyte ratio, glucose, HDL-c, and LDL-c and whether had hypertension, diabetes, and underwent PCI. Furthermore, multivariate adjusted linear regression models with bootstrap resampling also suggesting testosterone correlated significantly with GDF-15.

### GDF-15 and CAD

GDF-15 is a member of the transforming growth factor-β superfamily [15], it is widely distributed in endothelial cells, cardiomyocytes, and adipocytes. Due to paracrine/autocrine effects, levels of GDF-15 were upregulated by various cardiac stress and inflammation [16, 17]. Saskia et al. [18] have demonstrated that GDF-15 is progressively expressed in atherosclerotic lesions in a pattern similar to that of macrophages and GDF-15 deficiency protected against atherosclerosis by attenuating CCR2-mediated macrophage chemotaxis, indicating that GDF-15 knockout has a beneficial effect both in early and later atherosclerosis. Gabriel et al. [19] showed that GDF-15 was involved in orchestrating atherosclerotic lesion progression by regulating apoptotic cell death and IL-6-dependent inflammatory responses to vascular injury. These results indicate that GDF-15 is involved in the development of cardiovascular disease and it plays a crucial role in the pathogenesis of atherosclerosis.

In vitro, Kim et al. reported that the positive correlation between GDF-15 and C-reaction protein in a molecular level, which facilitated improved understanding of the pivotal inflammatory pathways important in CAD [20]. A recently published study observed an association of higher GDF-15 concentration with risk for mortality and heart failure in patients with CKD [21]. It indicated the association of GDF-15 with cardiac remodeling, which was the potentially important pathways in the pathogenesis of cardiovascular disease. Besides, serum GDF-15 levels were in relation to disease severity [22] and elevated GDF-15 levels were helpful in classifying high-risk ACS patients who benefit from high-dose highly efficient statins [23], as well as predicting CV‑death in a population of CAD patients after PCI [24]. And numerous studies [14] suggested that GDF-15 still added prognostic value to standard risk factors for predicting death, overall cardiovascular events, and provided additional information for risk stratification. All these data provide insight into the relationship between GDF-15 and CAD and supported that GDF-15 may be a specific marker for cardiovascular diseases, and this may be a particularly relevant pathway for inflammatory disease conditions such as CAD. Consistent with the previous studies, in our research, concentrations of GDF-15 in male patients with CAD were higher than controls, and GDF-15 levels increased with the severity of the disease.

### Testosterone and CAD

Testosterone is a vasoactive hormone responsible for vasodilatory effects on several vascular beds. It mainly involves the classical pathway which through binding to its receptor (AR) and subsequent genomic actions, and the non-genomic pathway which may be stimulated by exogenous androgen therapy. The means by which testosterone may confer benefit in the context of atherosclerosis were still remain poorly understood. Data from epidemiological statistics show that the concentration of circulating testosterone in men decreases with age [25, 26], in parallel with an accumulation of occurrence age-related diseases, such as CAD. The relationship between testosterone and cardiovascular disease (CVD) event has also been deeply excavated. A growing body of evidence supports that low circulating testosterone concentration has been correlated with accelerated vascular aging and reduced endogenous testosterone in middle-aged and older men are correlated with adverse cardiovascular outcomes, including a higher incidence of CAD and increased cardiovascular and all-cause mortality [27–29]. However, Dorte et al. reported that the event rate of CVD was higher in polycystic ovary syndrome (PCOS) compared to controls, which means testosterone levels in women with PCOS did not predict risk of cardiovascular disease and reminded us of paying attention to risk of developing CVD in young women with PCOS [30].

In the last decades, far more studies focusing on the individual effects of testosterone. And there is an ongoing debate in the medical community regarding the effects of testosterone on cardiovascular health, although the association between testosterone deficiency and CAD in men is confirmed in several large meta-analyses [31, 32]. Several studies have provided evidence that testosterone treatment with reduced risk of cardiovascular events or reduced cardiovascular mortality [33–35], and improved vascular function, characterized by arterial stiffness, large elastic artery stiffening (reduced compliance), intimal-medial thickening (IMT), and endothelial dysfunction [5, 36]. Whereas, a recently published paper has demonstrated that higher levels of testosterone associated with increased CVD and CAD. Andrew et al. summarized the current evidence regarding the relationship between testosterone (endogenous and supplemental) and cardiovascular health and suggested that normal physiologic levels of testosterone are beneficial to the male cardiovascular system and that testosterone deficiency is associated with an unfavorable metabolic profile and increased CVD events [37]. In our study, testosterone presented a inverse relationship with the severity of CAD, and testosterone levels in AMI patients were significantly higher than in SA, and UA patients.

### Testosterone and GDF-15

Yamazaki et al. reported that testosterone exhibits anti-inflammatory effects involving macrophages, as evidenced by suppressed mRNA levels for inflammatory cytokines such as MCP-1, TNF-α, and IL-6 [38]. In macrophages, GDF-15 may exert its immunomodulatory effects at a focal level within the plaque, directly alter intimal macrophage accumulation, apoptosis/necrosis, and collagen production, consequently resulting in distinct compositional differences of the atherosclerotic plaques [18]. On the other hand, testosterone might directly regulate the expression and secretion of GDF-15 in prostate cancer cells [9, 10, 39]. Therefore, decreased circulating testosterone levels may lead to the upregulation of GDF-15, either locally in the aortic tissues or systemically in the vasculature, and that may contribute to the progression of CAD.

Of note, our results demonstrated that CAD patients had high concentrations of GDF-15 and low levels of testosterone. Furthermore, the results of comparison means of GDF-15 across different testosterone categories showed that GDF-15 presented a strongly negative relationship with testosterone. It indicated that high GDF-15 levels may, at least in part, be attributed to testosterone deficiency. Moreover, these findings suggest that GDF-15 signaling is related to vascular injury and that GDF-15 signaling may have a crucial role in the context of testosterone deficiency. There is a potential interaction between high GDF-15 levels and testosterone deficiency in the development of CAD. Moreover, upregulation of GDF-15 association with testosterone provides novel perspective regarding the influence of testosterone on serum GDF-15 levels in patients with CAD.

The current study suffers from some limitations. First, it was a case–control study, which meant that it could only show associations, not causality. And more longitudinal studies and follow-up are required to confirm the correlation between GDF-15 and testosterone and verify the clinical significance. Second, Rovella et al. [40] has confirmed obesity is an independent risk factor for carotid plaque destabilization, particularly in males aged < 70 years. However, we did not take obesity into our consideration. Third, although we adjusted for multiple potential confounders, we cannot exclude the possibility of residual confounding. Finally, the sample size was small, further larger scale studies are required to confirm these results.

## Conclusion

In male patients with CAD, circulating GDF-15 levels are inversely associated with testosterone. This provide novel results regarding the influence of testosterone on serum GDF-15 levels, and upregulation of GDF-15 in the context of testosterone deficiency during CAD progression is a potential mechanism by which GDF-15 affects CAD.

## Additional files

**Additional file 1: Table S1.** Serum concentrations of GDF-15 and testosterone among SA, UA and AMI groups.

## Abbreviations

CAD: coronary artery disease
CVD: cardiovascular disease
GDF-15: growth differentiation factor-15
hs-CRP: high-sensitivity C-reactive protein
HDL-c: high-density lipoprotein cholesterol
LDL-c: low-density lipoprotein cholesterol
NEU: neutrophil
TC: total cholesterol
TG: triglycerides
PCI: percutaneous coronary intervention
WBC: white blood cell
