# Supplementary material for: Potential relation between soluble growth differentiation factor-15 and testosterone deficiency in male patients with coronary artery disease
Source: Cardiovasc Diabetol. 2019 Feb 28;18:21. doi: 10.1186/s12933-019-0823-3 (PMC6394066; doi:10.1186/s12933-019-0823-3)
Supplement: Supplementary file 1 — Additional file 1: Table S1. Serum concentrations of GDF-15 and testosterone among SA, UA and AMI groups. [file 12933_2019_823_MOESM1_ESM.docx]

**Table S1** Serum concentrations of GDF-15 and testosterone among SA, UA and AMI groups.

| Variables | SA | UA | AMI | *p* value |
| --- | --- | --- | --- | --- |
| GDF-15 | 545.05 (276.68—890.06) | 708.53 (373.96—1187.02) | 849.80 (538.04—1306.37) | 0.001 |
| testosterone | 337.33 (258.22—411.43) | 298.95 (227.32—384.78) | 285.86 (182.04—395.40) | 0.035 |

Data are expressed as median (25th percentile—75th percentile); and Kruskal-Wallis H test were performed to compare serum levels of GDF-15 and testosterone among SA, UA, and AMI groups.

*GDF-15* growth differentiation factor-15.
